# Infection of Mammals and Mosquitoes by Alphaviruses: Involvement of Cell Death

**DOI:** 10.3390/cells9122612

**Published:** 2020-12-05

**Authors:** Lucie Cappuccio, Carine Maisse

**Affiliations:** 1 IVPC UMR754 INRA, Univ Lyon, Université Claude Bernard Lyon 1, EPHE, 69007 Lyon, France; lucie.cappuccio@univ-lyon1.fr; 2 Interspecies Transmission of Arboviruses and Therapeutics Research Unit, Institut Pasteur of Shanghai, Chinese Academy of Sciences, Shanghai 200031, China

**Keywords:** alphaviruses, apoptosis, cell death, mosquito, tolerance

## Abstract

Alphaviruses, such as the chikungunya virus, are emerging and re-emerging viruses that pose a global public health threat. They are transmitted by blood-feeding arthropods, mainly mosquitoes, to humans and animals. Although alphaviruses cause debilitating diseases in mammalian hosts, it appears that they have no pathological effect on the mosquito vector. Alphavirus/host interactions are increasingly studied at cellular and molecular levels. While it seems clear that apoptosis plays a key role in some human pathologies, the role of cell death in determining the outcome of infections in mosquitoes remains to be fully understood. Here, we review the current knowledge on alphavirus-induced regulated cell death in hosts and vectors and the possible role they play in determining tolerance or resistance of mosquitoes.

## 1. Alphaviruses

Viruses belonging to the *Alphavirus* genus can be found in an ecological but not taxonomic group called arboviruses (an acronym for “arthropod-borne viruses” [1]). These viruses are transmitted by a hematophagous arthropod to a vertebrate host during a blood meal; in the case of alphaviruses, the predominant vectors are mosquitoes [2].

Alphaviruses are small, enveloped viruses of approximately 70 nm of diameter. The positive-sense, single-strand RNA contains two open reading frames (ORFs) that encode four non-structural proteins (nsp1–4) and five structural proteins (capsid, E3, E2, 6K, and E1) [3]. Alphaviruses include approximately 30 members, and infection results in clinical symptoms range from mild to severe [3]. Historically, alphaviruses were divided into New World and Old World alphaviruses, following their global distribution, evolution, pathogenicity, tissue, and cellular tropism or interactions with respective hosts. Old World alphaviruses (chikungunya virus (CHIKV); Sindbis virus (SINV); Semliki Forest virus (SFV); Ross River virus (RRV), etc.) were mainly found in Asia, Africa, and Europe, while New World alphaviruses (Eastern, Western, Venezuelan, and Equine Encephalitis Viruses (EEVs); Mayaro virus (MAYV)) were found in North and South America. However, with the global spreading of these viruses and their vectors, this division between New and Old World has become obsolete. Alphaviruses are now divided into three categories: aquatic viruses, arthritogenic viruses, and encephalitic viruses [4,5]. Infections by arthritogenic viruses in humans are characterized by rashes, fever, joint and muscle pain, and encephalitis for some of them (e.g., SINV, SFV). In some cases, incapacitating arthralgia and myalgia can last for months or years after infection (e.g., CHIKV, RRV, MAYV). Encephalitic virus infections are characterized by debilitating febrile disease and encephalomyelitis, leading to death in some cases (e.g., EEEV, VEEV) [3,6].

In mammals, skin cells are the first cells targeted by an arbovirus, such as an alphavirus, when inoculated by an infected mosquito. They are not clearly defined for each alphavirus but may be dermal fibroblasts [7], dermal dendritic cells, enterocytes or keratinocytes [8,9]—they constitute the first line of defense. Viruses will then reach other organs, such as joints, muscles [10] or the brain, where they will trigger pathology through induced cell death in the acute phase or long-lasting inflammation during the chronic phase [6].

In mosquito vectors, the arboviral infection is persistent and lasts the insect’s whole life. In comparison with their effect in the vertebrate host, alphaviruses do not seem to cause significant pathology in the mosquito vector. Even if some fitness costs have been described for some arboviruses [11,12,13], many other studies have concluded that arboviral infection is mainly silent and that mosquito vectors are tolerant to arboviruses [14,15]. From the oral acquisition of a viremic bloodmeal to the transmission to a new uninfected vertebrate host, alphaviruses replicate in arthropod cells and must cope with antiviral pathways. In mosquito, the first limit threshold to cross is the gut epithelium (i.e., midgut barrier), where the virus replicates to join the hemocoel (blood-containing body cavity) thus allowing viral spread to the whole body. To be transmitted again through blood feeding, the virus must penetrate the basal lamina of the salivary glands (salivary gland barrier) to join the acinar cells and replicate inside [16].

Interestingly, among the approximately 112 mosquito genera, the *Aedes* and *Culex* genera (such as *Aedes Albopictus*, *Aedes Aegypti*, *Culex Quinquefasciatus* or *Culex Pipiens*) seem to be the main vectors able to transmit viruses to humans [17]. Indeed, some mosquitoes may bite preferentially animals or may not be viral-transmission competent. As will be described below, part of the competence is linked to the different tissue barriers that can be crossed or not during the viral dissemination in the mosquito; this depends on innate immune response and cell death regulation in the infected cells [15,17,18].

## 2. Cell Death in Mammals

Cell death pathways can be divided into two opposite processes: accidental cell death (ACD) and regulated cell death (RCD). If ACD is a consequence of a severe and rapid injury (osmotic forces, pH variations, lytic viral replication), RCD is based on dedicated molecular machinery, implying that it can be modulated by pharmacological, genetic or infectious interventions [19].

Regulated cell death occurs under two different circumstances. The first one is programmed cell death (PCD) [20], which occurs during embryonic development or in the event of tissue homeostasis. The second one regroups different RCD pathways that occur following an external or internal, prolonged, and intense stress event. This contributes to tissue homeostasis and protection by eliminating useless or potentially dangerous cells (i.e., malignant or infected cells).

Dying cells present different and well described morphological features that have been used so far to classify cell death processes into three main types [19]: apoptosis, autophagy-dependent cell death, and necrosis. Apoptosis is characterized by chromatin condensation, nuclear fragmentation, cytoplasmic shrinkage, membrane blebbing, and the formation of “apoptotic bodies”, which are subsequently destroyed by professional or non-surrounding phagocytes. Autophagy-dependent cell death is essentially defined by its distinctive features of extensive cytoplasm vacuolization, leading to phagocytosis and degradation by lysosomes. Necrosis is mainly characterized by swelling, plasma membrane disruption, and cytoplasmic content efflux in the extracellular environment, without evident phagocytosis or lysosomal degradation by the neighboring cells.

Intuitively, necrosis is associated to ACD, but it is nowadays clear that some RCD can also lead to non-apoptotic cell death as recently described in necroptosis [21], pyroptosis [22], and ferroptosis [23].

We will focus here on the cell death pathways that have been shown to be involved in antiviral response so far, without considering subtypes such as attachment dependence (i.e., anoïkis) and entotic cell death, parthanos, etc. For complete reviews see References [19,24].

### 2.1. Intrinsic Apoptosis

“Intrinsic apoptosis is a form of RCD initiated by perturbations of the intracellular or extracellular microenvironment, demarcated by mitochondrial outer membrane permeabilization (MOMP) and precipitated by executioner caspases, mainly caspase 3 (CASP3)” (Nomenclature Committee on Cell Death (NCCD) [19]). It can be induced by numerous dysregulations including DNA damage, endoplasmic reticulum (ER) stress, reactive oxygen species (ROS) overload or infection. The main characteristic is that cells still present plasma membrane integrity and metabolic activity, leading, in vivo, to the removal of apoptotic bodies by surrounding phagocytic cells that recognize phosphatidylserine (PS) at the cell surface. In vitro, unless the cultured cells present phagocytic capacities, apoptosis usually ends by a “secondary necrosis”, exposing damaged plasma membrane [25].

The decisive step of intrinsic apoptosis is the irreversible and extensive MOMP, leading to the release of numerous pro-apoptotic factors contained in the intermembrane space [26]. Mitochondrial outer membrane permeabilization is controlled by a family of 20 pro- or anti-apoptotic proteins: the B cells lymphoma 2 (Bcl-2) family proteins, which share one to four Bcl-2 homology domains (BH1 to BH4) [27]. All of them are finely regulated at the transcriptional and/or post-translational level (degradation, phosphorylation, localization, oligomerization, etc.) in order to integrate the extracellular or intracellular signals, which will potentially lead to apoptosis.

In mammals, only three of them (Bax, Bak, and Bok) have been described as able to form pores in the mitochondrial outer membrane (MOM) and other cellular membranes after oligomerization. These proteins are thus considered as “effectors” that can be activated, transcriptionally or post-translationally, after a cellular stress to induce MOMP [28,29]. Moreover, a pool of BH3-only proteins, described as “activators” promotes MOMP induction by interacting with Bax and Bak, allowing the conformational changes necessary for pore formation. These proteins can be post-translationally modified (e.g., Bid, cleaved in the pro-apoptotic truncated form “t-Bid”) [30,31] or transcriptionally activated (e.g., PUMA, Noxa and Bim). In particular, the transcription factor p53 represents one of the links between DNA damage or oxidative stress and intrinsic apoptosis. Indeed, after a stress signal, post-translational modifications induce p53 stabilization and translocation in the nucleus. p53 will induce pro-apoptotic Bcl-2 family proteins transcription (i.e., Bax, Bak, PUMA, and Noxa) [32,33]. In the absence of stress conditions, other members of the Bcl-2 family (Bcl-2, Bcl-Xl, Bcl-W, Mcl-1, and Bfl-1) constantly block MOMP [34]. They contain all four BH domains and are inserted in the MOM or ER membrane, interacting with and inhibiting the effectors members (Bax, Bak, and Bok) or the BH3-only activators (PUMA, Noxa, Bim, and tBid) [35,36]. In addition, these anti-apoptotic proteins have been shown to regulate Ca^2+^ homeostasis in the ER [37,38] and cellular redox equilibrium [39,40]. Finally, it has been shown that some BH3-only proteins (Bad, Bmf, and Hrk) carry out their pro-apoptotic effect without interacting with the effector proteins but by inhibiting the pro-survival ones [41].

Interestingly, it is now clear that mitochondria and ER are physically connected, forming platforms called mitochondria associated (ER) membranes (MAMs) [42]. Mitochondria associated membranes regulate numerous cellular processes such as calcium (Ca^2+^) homeostasis, autophagy, lipid metabolism, apoptosis, and the rapid exchange of biological molecules [43]. They are involved in inflammasome formation and activation and participate in the antiviral response through the mitochondrial antiviral protein (MAV)/RNA sensors (retinoic acid-inducible gene I (RIG-I) or melanoma differentiation-associated protein 5 (MDA5)) complex activation [44].

Mitochondrial outer membrane permeabilization induces the release of intermembrane space elements, among which cytochrome C (CYC), Omi, and DIABLO (also called SMAC) [45,46,47]. Furthermore, following MOMP, the mitochondrial transmembrane potential (ΔΨm) is usually lost, mostly due to CYC release in the cytosol and the consequent stop of the respiratory chain [48].

**Figure 1:**
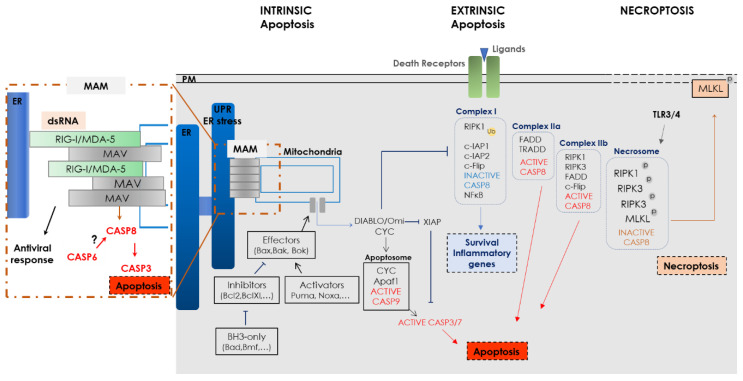
Apoptosis and necroptosis in mammals. PM: plasma membrane, ER: endoplasmic reticulum, MAM: mitochondria-associated membranes, CYC: cytochrome c, IAP: inhibitors of apoptosis proteins, RIPK1/3: receptor interacting serine/threonine kinase 1/3, Ub: uiquitin, p: phosphorylation, MLKL: mixed-lineage kinase domain-Like, FADD: Fas-associated protein with death domain, TRADD: tumor necrosis factor receptor super family (TNFR1)-associated death domain protein, TLR3/4: Toll-like receptor.

In the cytosol, the association of CYC with Apaf1 and pro-caspase 9 (CASP9) forms a complex called apoptosome that will activate CASP9 in an ATP-dependent process [45]. In turn, the activated CASP9 will then activate the executioner caspases (i.e., mainly CASP3 and -7) that are involved in the final cellular destruction: poly (ADP-ribose) polymerase (PARP) cleavage, DNA fragmentation, PS exposure, apoptotic bodies formation [49,50,51,52]. Omi and DIABLO enhance cell death by inhibiting the inhibitor of apoptosis protein (IAP) family, which includes XIAP, c-IAP1, and c-IAP2. XIAP is constitutively bound to executioner CASP3 and -7 and, thus, blocks their activity [53,54]. c-IAP1 and c-IAP2, for their part, are two E3 ubiquitin ligases. They upregulate the CASP8 inhibitor c-Flip, induce caspases’ degradation through ubiquitination, and promote NF-κB pro-survival pathway through receptor interacting serine/threonine kinase 1 (RIPK1) ubiquitination. These functions have mostly been maintained throughout evolution, from insects to mammals [55,56,57] (Figure 1).

Finally, executioner caspases can positively or negatively regulate the emission of multiple damage-associated molecular patterns (DAMPs) by dying cells, including immunostimulatory [58] as well as immunosuppressive [59] factors.

### 2.2. Extrinsic Apoptosis

“Extrinsic apoptosis is a type of RCD initiated by perturbations of the extracellular microenvironment that are detected by plasma membrane receptors, propagated by CASP8 (with the optional involvement of MOMP), and precipitated by executioner caspases, mainly CASP3” (NCCD [19]).

Extrinsic apoptosis is mainly carried out by the activation of two main receptors types: death receptors and dependence receptors.

Dependence receptors are a functional family of around 20 receptors, characterized by the induction of a positive signal when bound by their ligand (survival, proliferation, differentiation, etc.) while they activate RCD in the absence of the ligand. Among them can be found netrin-1 receptors (deleted in colorectal carcinoma (DCC) [60], uncoordinated 5 homologs (UNC5Hs, UNC5H1,2,3,4 also called UNC5A,B,C,D) [61], the neogenin receptor [62], the low affinity neurotrophin receptor, p75 neurotrophin receptor (p75NTR) [63], and receptors with tyrosine kinase activity (e.g., rearranged during transfection (RET) [64], tropomyosin receptor kinase A and C (TrkA and TrkC) [65], and c-kit (CD117) [66]). Their physiological role is mainly cell guidance and they are mostly involved in tumor progression when dysregulated [67]. Even if Netrin-1 plays a role in inflammation regulation [68,69,70,71], dependence receptors have not been involved, so far, in antiviral response [67]. As such, they will not be described in this review.

Death receptors include Fas (CD95, APO-1), the tumor necrosis factor receptor super family TNFRSF1A (TNFR1), 10a (TNF-related apoptosis-inducing ligand receptor TRAILR1, DR4), and 10b (TRAILR2, DR5) [72,73]. The general mechanism is that ligand binding induces the receptors’ oligomerization and the subsequent recruitment through their death domains (DDs), of adapter proteins in the intra-cellular side, to form a “death-inducing signaling complex” (DISC).

Fas ligand or TRAIL binding drives the oligomerization of their receptors, the recruitment of Fas-associated protein with death domain (FADD) through the DD and the subsequent formation of the DISC through interaction with CASP8 via death effector domain (DED) and different isoforms of cFlip [74,75].

Bound TNFR1 interacts with TNFR1-dssociated death domain protein (TRADD), through its DD, which enables the formation of “Complex I”. The subsequent formations of “Complex II” (“IIa” or “IIb”) operate as molecular platforms to regulate the activation and functions of CASP8 (or CASP10, in some cases) [76,77]. CASP8 activation leads to RCD following two different pathways. In Type I cells, CASP8 directly activates CASP3 and -7 thus inducing the execution of the apoptotic pathway. In Type II cells, where CASP3 and -7 are sequestrated by XIAP, extrinsic apoptosis occurs through the cleavage by CASP8 of Bid, a BH3-only protein, and the release of its truncated form, t-Bid. t-Bid acts as an activator on Bax and Bak to provoke MOMP and the subsequent CASP9-dependent RCD, described above in intrinsic apoptosis [78].

CASP8 activation is the key process of extrinsic apoptosis; its regulation is complex and also involved in inflammation and antiviral response [77]. cFlip is one of the key components that promotes or inhibits CASP8 oligomerization and ensuing activation by autoproteolytic cleavage. As cFlip is transcriptionally regulated by NF-κB, it can also participate in a pro-survival pathway induced by TNFR1 in some conditions [79]. It is indeed increasingly clear that the activation of death receptors by their ligands does not necessarily lead to RCD but can also activate pro-survival signals. Specifically, the TNFR1-induced pathway depends on the RIPK1 ubiquitination level, which directly influences the formation of pro-survival versus pro-death complexes.

Briefly, in Complex I, RIPK1 polyubiquitination by cIAP1 and cIAP2 leads to NF-κB activation, pro-survival, and inflammatory genes transcription, where a high level of cFlip is correlated to survival. Subsequently, deubiquitinated RIPK1 is released from Complex I and forms Complex IIa in the cytosol with FADD, TRADD, cFlip, and CASP8. If cFLIP concentration is low, this complex leads to the degradation of RIPK1 and RIPK3, allowing CASP8 dimerization and activation and the subsequent apoptotic cell death through CASP3. In a context of high cFlip concentration, CASP8/cFlip heterodimers are formed and apoptosis is blocked [56,57,78]. Moreover, in the absence of cIAP (after MOMP and IAP inhibitors release for instance), phosphorylated RIPK1 leads to non-canonical NF-κB activation and subsequent association with RIPK3, FADD, and cFlip to activate CASP8 (Complex IIb) [73].

Finally, another possible pathway is induced when CASP8 is inhibited by chemical caspase inhibitors or virally encoded proteins. In this case, deubiquitinated RIPK1 and RIPK3 bind in microfilaments, “amyloid-like” complexes called necrosomes (most likely trimers or tetramers) [80,81]. The auto- and transphosphorylation of RIPK1 and RIPK3 and the recruitment of mixed lineage kinase domain-Like (MLKL) to the plasma membrane, triggering membrane permeabilization, initiate what is called necroptosis [82]. It is of interest to note that MLKL oligomers also lead to PS exposure, a feature usually considered as a hallmark of apoptosis [80].

If CASP8 is inhibited, RIPK3 phosphorylation can be triggered by some activated PRR (pathogen recognition receptors), such as TL3 and TL4 [83], nucleic acid sensors, such as RIG-I and MDA5, and some adhesion receptors [84,85]. In addition, IFNα and β receptor subunit 1 (IFNAR1) and IFNγ receptor 1 (IFNGR1) are also able to trigger necroptosis through TRIF and ISGF3 activation [86] (Figure 1).

### 2.3. Inflammasome Activation and Pyroptosis

“Pyroptosis is a form of inflammatory RCD that critically depends on the formation of plasma membrane pores by members of the gasdermin (GSDM) protein family, often as a consequence of inflammatory caspase (CASP1, 4 or 5) activation” (NCCD [19]).

Pyroptotic cells present PS exposure, chromatin condensation, TUNEL staining but no DNA laddering, and a slight MOMP. Final GSDM-dependent membrane permeabilization allows the release of pro-inflammatory cytokines (IL1β and IL18, both NF-κB-target genes), maturated by interleukin-1β-converting enzyme (ICE/CASP1)-dependent cleavage. Other factors are also released, thus participating in the defense against pathogens through inflammation and the induction of an adaptive response [19,87,88]. In fact, pyroptosis seems to be mainly involved in the innate immunity against intracellular pathogens [88]. Pyroptosis was first thought to be restricted to monocyte/macrophage lineage, but it has been observed in other cells [89].

**Figure 2:**
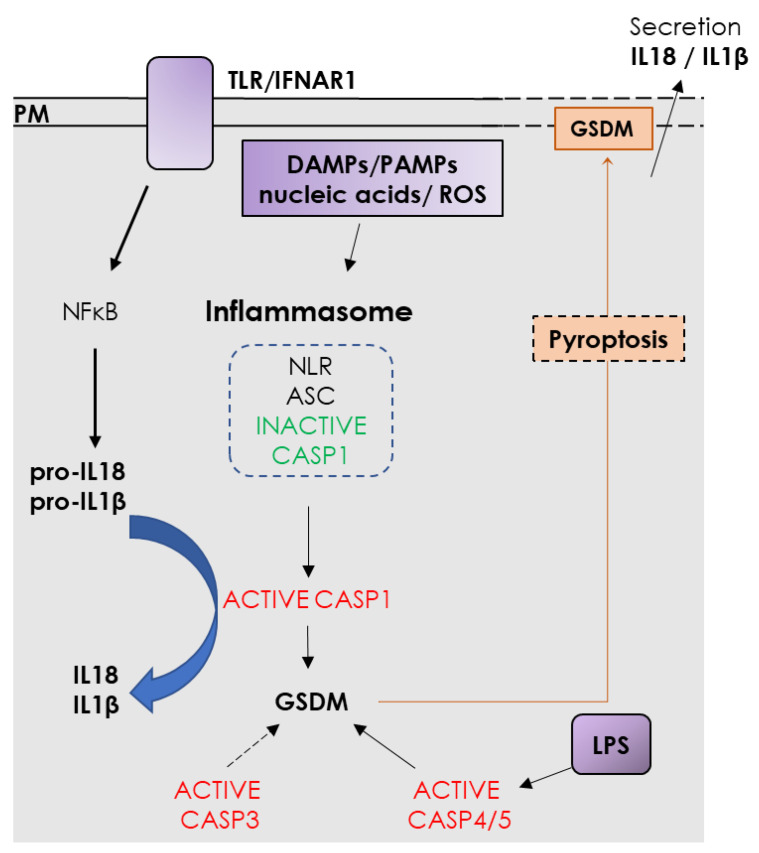
Inflammasome activation and pyroptosis. PM: plasma membrane, TLR: Toll-like receptor, IFNAR1: Interferon associated-receptor 1, DAMP: damage-associated molecular patterns, PAMPs: pathogen-associated molecular patterns, ROS: reactive oxygen species, NLR: NOD-like receptor, IL: interleukin, LPS: lipopolysaccharide.

The inflammasome is activated by different DAMPs or pathogen-associated molecular pattern (PAMPs). It is a multiprotein complex that, like the apoptosome (intrinsic apoptosis) or the DISC (extrinsic apoptosis), acts as a caspase-activating platform. It is formed by a receptor (NOD-like receptor (NLR) family or non-NLR (AIM2)), an adapter protein (ASC, apoptosis-associated speck-like protein containing a CARD) and CASP1 that cleaves pro-IL18, pro-IL1β and GSDM. However, it is now clear that pyroptosis can also be activated by other caspases such as CASP3 [90] (Figure 2).

The recent description of necroptosis and pyroptosis processes has amplified the complexity of RCD understanding. Contrary to general consensus so far, it is now clear that intrinsic as well as extrinsic regulated cell death can be immunogenic, thus participating in the establishment of the adaptive immune response [91,92]. This has been recently underlined by the description of the concomitant activation of apoptosis, necroptosis, and pyroptosis in a context of bacterial or viral infection in macrophages, leading to inflammatory cell death. The phenomenon has been named PANoptosis and would involve molecules of the three RCD pathways (i.e., CASP8, RIPK3, and CASP1) in a single complex called PANoptosome [93].

### 2.4. Autophagy-Dependent Cell Death

Autophagy-dependent cell death is a type of RCD that depends on components of the macroautophagy machinery [19]. Macroautophagy is a particular form of autophagy where double-membrane vesicles (autophagosomes) sequester a large part of organelles and cytoplasm, leading to their lysis and, in some cases, to cell death. Morphologically, dying cells present an accumulation of autophagosomes and autolysosomes in the cytoplasm, a feature extremely different from apoptotic or necrotic RCD. However, it seems increasingly clear that autophagy usually inhibits rather than induces cell death and has to be considered as a way for the cell to maintain homeostasis after stress signals (hypoxia, ROS, starvation, PRR activation, etc.) [94]. Even if in some cases inhibition of specific autophagy proteins can delay RCD [95], pharmacological or genetic inhibition of macroautophagy components usually accelerates the death of cells rather than protects them. Autophagy may degrade damaged mitochondria or pro-apoptotic complexes thus preventing cell death.

It is of interest to note that autophagy leads to the degradation by lysosomes of components of endogenous or exogenous origin, which are accessible in the cytoplasm. This has to be distinguished from vesicular trafficking, which starts in the plasma membrane and also leads to lysosomal degradation (i.e., phagocytosis or receptor-mediated endocytosis). Macroautophagy and vesicular trafficking pathways interact at numerous regulation points, especially in the late phases of the pathways. Autophagosomes or late endosomes fusion with lysosomes actually require the same machinery. Thus, numerous proteins involved in physiological or lytic autophagy are also essential for viral penetration, from receptor-mediated endocytosis to fusion of the viral envelope with the endosome membrane and subsequent liberation of the viral genome in the cytosol.

## 3. Impact of Alphaviral Infection on Regulated Cell Death

During a viral infection, RCD is generally described as a defense mechanism, induced to limit virus replication and production, to prevent infection of neighboring cells and, to some extent, to participate in immune response induction. Cell death induced by alphavirus infection has been observed and studied in several cell types infected by different alphaviruses, mainly CHIKV, SFV, SINV, VEEV, and EEV.

### 3.1. Apoptotic Pathways in Alphavirus-Infected Cells

Infections of baby hamster kidney (BHK), rat prostatic adenocarcinoma (AT-3), and mouse neuroblastoma (N18) cells with SINV result in clear nuclear condensation and membrane blebbing, 24 h post-infection [96]. In the same context, SFV infection of AT-3 induces apoptotic features, correlated to strain virulence. Grandgirard et al. [97] have described, in rat embryonic fibroblasts, a potential caspase-dependent Bcl-2 cleavage in SFV- or SINV-infected cells leading to cell death and viral replication, even in a context of Bcl-2 overexpression. In 293T and BHK cells, the BH3-only protein Bad seems to participate in SINV-induced cell death, through its interaction with some specific anti-apoptotic Bcl-2 proteins, while the other non-binding members also regulate cell death [98]. The dynamics of mitochondria are also highly altered during apoptosis induced by VEEV infection of human astrocytoma cells U87MG [99]. Infection rapidly induces MOMP and ROS increase, followed by perinuclear localization and fission of mitochondria, and then mitophagy. Moreover, VEEV capsid co-localizes with mitochondria and could participate in mitochondria dysregulations.

Lin et al. showed a link between SINV induced cell death, oxidative stress, NF-κB, and Bcl-2 expression. In AT-3 and N18 infected cells, NF-κB activation and cell death were indeed inhibited either by antioxidant agents or Bcl-2 overexpression, with no effect on viral entry or replication however [100]. Likewise, MRC5 human fibroblasts present a SINV persistent infection, when manganese-superoxide dismutase (Mn-SOD) is over-expressed, confirming that oxidative pathways are implicated in the effects of SINV [101]. Chikungunya virus infection has been studied in the neuroblastoma cell line SH-SY5Y by Dhanwani et al. [102]. Intrinsic apoptosis features (CYC release, CASP3 activation, PARP cleavage) are observed 24 h and 36 h post-infection. Moreover, the infection is followed, 36 h and 48 h after by an elevation of ROS, a decrease of anti-oxidant enzymes expression and glutathione (GSH) depletion.

Another cellular response to stress induced by viral infection is the unfolded protein response (UPR) of the ER due to the accumulation of newly synthetized viral proteins in the ER, leading to translation blocking and intrinsic apoptosis [103]. Endoplasmic reticulum stress response has been described in *flavivirus*-infected cells, but little is known about alphaviruses. However, SFV envelope glycoproteins, but not capsid, seem able to induce and accelerate apoptotic cell death [104], while VEEV glycoproteins induce UPR and apoptosis in primary astrocytes [105,106].

Finally, intrinsic apoptosis may be triggered by alphavirus non-structural protein activity. Indeed, SINV, VEEV, and EEEV nsp2 and nsp3 have been shown to be responsible for viral cytopathic effects, enabling persistent infections when specifically mutated [107,108,109]. Frolov and colleagues have described the nuclear translocation of nsp2 and global transcriptional shutoff through RNA polymerase II degradation [110,111,112] and a subsequent nsp3-dependent translational shutoff for arthritogenic viruses [112], while capsid would play this role for encephalitic viruses [113]. However, CHIKV nsp2 seems to inhibit UPR as well through its transcriptional shutoff activity [114] and to interfere with the IFNβ signaling pathway [115,116].

However, Sarid et al. characterized a CASP8 and TNFα/TNFR1-dependent PC-12 RCD after infection by the SINV SVNI strain (neurovirulent and cytotoxic). Indeed, they described an upregulation of TNFα expression in infected cells and a cell death inhibition following cFlip overexpression [117]. Nava and colleagues [118] have shown, in SINV-infected BHK cells and in mice, an inhibition of death after treatment with the pan-caspase inhibitor zVAD-fmk and the CASP1 and CASP8 inhibitor CrmA (a serine proteinase inhibitor from Cowpox virus). In addition, by using SFV replicon vectors or a wild-type SFV strain, Kiiver et al. have neither shown any Bcl-2 protective action against virally induced cellular protein synthesis shutdown post-infection nor cell death. Moreover, AT-3 and BHK cells did not present any CYC release after infection [119]. In addition, unlike poxvirus, that blocks CASP1 and CASP8 [120,121], or herpesviruses [122], alphaviruses have never been described as inducing necroptosis.

Thus, on the one hand, alphaviruses appear to induce apoptosis through mitochondria, oxidative, and ER stress or the transcriptional and translational shutoff induced by nsp (intrinsic pathway). On the other hand, some studies suggest the implication of death receptors and CASP8 activation, without mitochondrial involvement (extrinsic pathway). This apparent discrepancy may be explained by the different apoptotic cells that have been observed (infected or neighbor cells) and the duration of infection (few hours to days post-infection). Indeed, Joubert et al. suggest that, in a first wave, CHIKV-infected cells die through intrinsic apoptosis (CASP9 positive cells) and that, in a second time, during antiviral response, infected, and neighbor cells die through the extrinsic pathway (death receptors and CASP8 activation). These secondary pathways seem to be independent of ER and oxidative stress [123,124].

Other explanations could be found also in two recent studies, which involve two newly described pathways in SFV-induced cell death. Using 3T9 MEFs, Urban et al. [125] characterized SFV-induced RCD. In their study, they first showed that RCD was triggered by SFV replication and not only by viral entry, as previously described [126]. Secondly, SFV-induced cell death occurred in a Bak dependent MOMP, leading to apoptosome activation. Moreover, they excluded the involvement of TRAILR, Fas or TNFR1 in this process. Surprisingly, CASP8 and tBid seemed to be activated downstream of apoptosome, maybe through CASP6 [127], acting as an amplification loop. In fact, although CASP6 has long been considered as an executioner caspase based on its homology with CASP3 and CASP7, recent data suggest that CASP6 may actually be involved in RCD initiation [127,128]. Additional investigation is required to elucidate the function of CASP6 in mammalian cells. Secondly, in several SFV-infected cell types, El Maadidi et al. recently described a new mitochondrial platform, involving the innate immune factor MAV and the initiator CASP8, comparable to the death-inducing signaling complex (DISC) of the death receptors signaling. However, this complex does not involve FADD but another potential, not yet characterized adaptor. The platform is activated via the dsRNA sensors MDA5 or RIG-I and acts in parallel of the classical Bax/Bak dependent MOMP, also leading to CASP3 activation, independently of type I IFN signaling factors (IRF3, IRF7, IFNβ, PKR, etc.) and mitochondrial depolarization [129]. As MAVs platforms have been described in MAMs, it is tempting to hypothesize that the MAV/CASP8 should be localized in these subcellular structures thus interacting with other metabolic pathways (such as ROS, Ca^2+^, lipid, autophagy) during antiviral response [130].

Hence, it appears that several alphaviruses induce regulated cell death in numerous cell types, involving mitochondria depolarization, ER stress, and CASP8 activation, maybe in a time-dependent regulation or through alternative processes. However, after nearly 30 years of study, large parts of the molecular pathway leading an infected cell to death remain to be deciphered. It would be of interest in the future studies to focus on the cell types relevant for the viral tropism (e.g., skin cells, muscle cells, neural cells) and to favor innovative cell culture technics that mimic better the natural cell characteristics (e.g., 3D culture, explants, iPS (induced Pluripotent Stem cells)).

### 3.2. Inflammasome and Pyroptosis in Alphaviral Infection

Activation of the inflammasome pathway and pyroptosis has been intensively studied for flaviviruses infection, especially for their involvement in pathogenesis [131,132,133], but little is known for alphaviruses. Even if inflammasome pathways seem to participate in the pathology and the response against alphaviruses, the involvement of pyroptosis has never been described. In dermal fibroblasts, CHIKV and the *flavivirus* West Nile virus (WNV) both induce IL1β production and CASP1 activation through the AIM2 inflammasome sensor, but only CHIKV replication and propagation can be controlled by CASP1 [134]. In PBMC from CHIKV-infected patients, high levels of NLRP3, IL18, and CASP1 are found [135]. Moreover, in mice, NLRP3 activation is correlated to inflammatory symptoms such as bone damage and myositis. NLRP3 inhibition leads to a reduction of the inflammatory pathology induced by CHIKV but not by WNV [135]. The alphavirus Mayaro (MAYV) induces the expression of inflammasome proteins in macrophages, and inflammatory cytokines production through the NLRP3 sensor, activated by ROS and K^+^ efflux. In mice, NLRP3 is also involved in MAYV induced pathogenesis [136].

Thus, inflammasome activation has been mainly involved in global inflammatory response to alphavirus in vivo, but the molecular pathways activated in the cell remain to be described. Indeed, it is still unknown if pyroptosis may participate in inflammatory cytokines secretion during alphavirus infection, and, to our knowledge, there is no molecular study of this process.

## 4. Interplay between Cell Death and Alphaviral Replication and Spread in Mammals

Apoptosis appears to be a strong antiviral process. Indeed, Bcl-2 overexpression converts SINV infection from lytic to persistent in vitro [96] and in vivo [137]. Moreover, Bcl-2 seems to be able to restrict SFV replication by inhibiting early stages of infection and appears to prolong survival of productively infected cells [138].

As described above, autophagy usually blocks apoptosis, and viruses have developed strategies to take advantage of this property. The first connection between alphavirus infection and autophagy has been made by Liang and colleagues [139], when they identified Beclin-1 as a new Bcl-2-interacting protein through a yeast two-hybrids screening. Beclin-1 is a major factor of autophagy, involved in autophagosome initiation and maturation. In this study, Beclin-1 protected SINV-infected mice against fatal encephalitis, with a significantly lower viral replication rate in mice brains. The author correlated these observations with the previously observed protective role of Bcl-2 against in vivo SINV infection [96,137]. However, years after this study, Bcl-2 was described as a Beclin-1 inhibitor, thus participating in ER stress connected autophagy regulation [140]. Another crucial protein of the autophagy pathway, Atg5, protects mouse neurons from SINV-induced cell death [141], with no apparent impact on viral replication. Moreover, the adaptor protein p62 seems to be linked to viral capsid clearance by direct interaction and target of autophagosomes, thus promoting cell survival [141]. Finally, in SFV-infected cells, autophagosomes accumulate but autophagy modulation has no effect on viral replication, and this autophagosomes accumulation seems to be due to the inhibition of their degradation rather than an induction by SFV infection [142].

In HEK293 cells, CHIKV infection induces autophagy features (LC3 positive vesicles and electron microscopy observation). In this study, autophagy has a clear pro-viral role, increasing the number of infected cells and viral RNA in the cell culture supernatant [143]. Moreover, in vitro and in vivo, CHIKV infection has also been shown to induce an autophagy flux, through ER and oxidative stress [123,124]. In these models, autophagy limits (i) extrinsic and intrinsic RCD induced by CHIKV infection, (ii) mice lethality, and (iii) viral propagation. Autophagy, as a host response to infection, limits indeed the cytopathic effects of CHIKV and regulates the pathogenesis of acute chikungunya disease. However, during late phases of in vitro infection (48 h post-infection), a switch between autophagy and apoptosis is observed and cells die. Finally, in HeLa cells, autophagy promotes CHIKV infection and inhibits cell death. Indeed, in addition to p62-dependent capsid clearing, another autophagy receptor, NDP52, interacts with nsp2, localizes near the CHIKV replication complex and restricts cell shutoff thus promoting viral replication and cell survival [144].

Hence, alphaviruses may exploit autophagy to delay cell death through (i) direct inhibition of intrinsic and extrinsic apoptosis and (ii) a limitation of viral proteins production, allowing cell survival and a longer viral replication.

However, several Old World Alphaviruses, such as CHIKV, SFV, and RRV, seem able to activate the phosphatidylinositol-3-kinase (PI3K)–AKT–mTOR pathway, involved in cell survival and autophagy inhibition. Furthermore, inhibition of this pathway has a negative effect on viral replication [145]. This apparent discrepancy with the previous observations may indicate that, more than autophagy per se, cell survival is the key process which favors viral replication.

Finally, it is of interest to note that, in some cases, apoptosis has been shown to enhance viral spread. Indeed, in their study, Krejbich-Trotot and colleagues [146] first confirmed the dual nature of the alphavirus-induced apoptosis (intrinsic and extrinsic) in HeLa and primary fibroblasts infected with CHIKV accompanied by CASP8 activation in neighbor cells. More interestingly still, inhibitors of blebbing or engulfment drastically reduced infection rates. Finally, they detected infective CHIKV in apoptotic corpses and in the macrophages which phagocyted them, leading to macrophages infection and viral production. As macrophages are refractory to CHIKV infection in vitro, this study highlights a possible role of apoptotic blebs in viral propagation. This phenomenon, called “apoptotic mimicry”, is used by a large number of viruses to exploit the PS receptors present on numerous cells membranes, enhancing viral spread and limiting immune response [147].

## 5. Impact of Apoptosis on Virus Pathogenesis in Mammals

### 5.1. Alphavirus Encephalitis

The first cell death analysis was documented in vivo, using SINV, VEEV, and SFV, three alphaviruses causing encephalitis. In SINV-infected mice, the apoptotic cells were detected principally in the brain and contained viral antigens, suggesting that apoptosis was correlated to neurovirulence [148]. The in vivo mouse infection of VEEV was also associated to cell death in brain, demonstrated by TUNEL assay (DNA fragmentation) and morphological changes [149].

Comparing SINV infections with SVNI (neurovirulent and cytotoxic) or SVA (avirulent and leading to persistent infection) strains in PC-12 cells and astrocytes, revealed that SVNI induces Bax overexpression while SVA induces Bcl-2 expression [150].

Intranasally SFV-infected rats develop encephalitis, where infiltrating leucocytes and neural precursor cells undergo apoptosis while productively infected neurons present necrotic features, apparently due to the local inflammation [151].

Hence, it appears that alphaviruses pathogenicity is linked to its cytopathic effects in infected cells, at least in the case of the encephalitic group.

### 5.2. Alphaviral Chronic Infection: What about Cell Death?

One characteristic of the *Alphavirus* genus is the ability of some of them (CHIKV, SINV, MAYV, RRV, etc.) to induce chronic pain, such as arthritis and myalgia, which may last for years, with detectable viral genome in the organism. This persistent infection implies that some cells may be chronically infected, and in some way able to delay or block cell death. However, despite an intense immune response observed in chronic patients, damaged synovial tissues present strong apoptosis features. Chikungunya virus has been found in synovial macrophages several months after infection but joints do not seem to be the viral reservoir [152]. In addition, RRV-infected human monocyte acute leukemia MM6 cell line presents very low replication rates, without innate immune control, and apoptosis features at late stages of infection. This indicates that monocytes could be persistently infected and participate in the chronic form of RRV or CHIKV [153]. Young et al. [154] propose that dermal and muscular fibroblasts, as well as myofibers, may survive the acute CHIKV infection and harbor persistent CHIKV RNA during chronic phase of the disease. Moreover, they observe that synovial cells are not infected in large numbers in vivo and suggest that synovial cells may be infected but do not survive.

How these cells survive remains to be understood. The mechanisms involved in alphaviral persistence are mostly unknown. They may depend on infected cell type and the highly complex interplay between virus, immune response, and different RCD pathways.

## 6. Interplay between Cell Death and Susceptibility of Mosquito Species to Arboviruses

The interplay between arbovirus and arthropods is still poorly understood and the primary point of study concerns flaviviruses (mainly Dengue virus (DENV)) in mosquito and/or in *Drosophila*, used as a genetic model for insect immunity. Hence, very little is known concerning the impact of alphavirus infection in mosquitoes and which factors may explain the tolerance versus resistance of different mosquito strains.

It is classically admitted that alphaviruses do not induce any major pathology in their vectors. However, several lesions are observed in tissues which are critical for viral propagation and transmission. Indeed, after feeding on infected blood, cellular response in the midgut plays a decisive role in vector competence. EEEV infection of *Culiseta melanura* mosquito induces severe lesions in midgut epithelial cells and basal lamina, associated to viral spread [155]. Likewise, infection of more or less susceptible *Culex tarsalis* strains with WEEV revealed lesions and apparent necrotic cell death only in the sensitive mosquito’s gut [156]. Transcriptomic analysis of *Aedes Aegypti* fed with CHIKV in blood or different buffers reveals the over-expression of matrix metallo proteinases (MMP) and other peptidases in the midgut, as well as the decrease of Collagen IV, a component of the basal lamina [157]. Intrathoracically SINV-injected *Aedes Albopictus* present colocalization of virus antigen with structural lesions and TUNEL positive cells in salivary glands [158] and midgut-associated visceral muscles [159]. Furthermore, organ-associated muscles respond differently to SINV [160]: 10 days post-infection, the virus has cleared from the midgut, is persistent in the hindgut, and unable to infect ovary associated muscle cells. High viral titers induce pathology limited to gut associated muscles and gut epithelium. Finally, in *Aedes Aegypti* mosquitoes, AeIAP1 (IAP ortholog) downregulation leads to a higher replication of SINV in the midgut, while AeDronc (CASP9 ortholog) inhibition is associated to a lower viral replication and dissemination towards salivary glands [161] (see Box 1 for RCD pathway description in insect).

Box 1 **Comparative cell death pathways in mammal, *Drosophila* and *Aedes.* PM: plasma membrane; CYC: cytochrome C; IAP: Inhibitors of apoptosis; *Ae*: *Aedes*; RHG: Reaper, Hid and Grim; IMD: immune deficiency.**

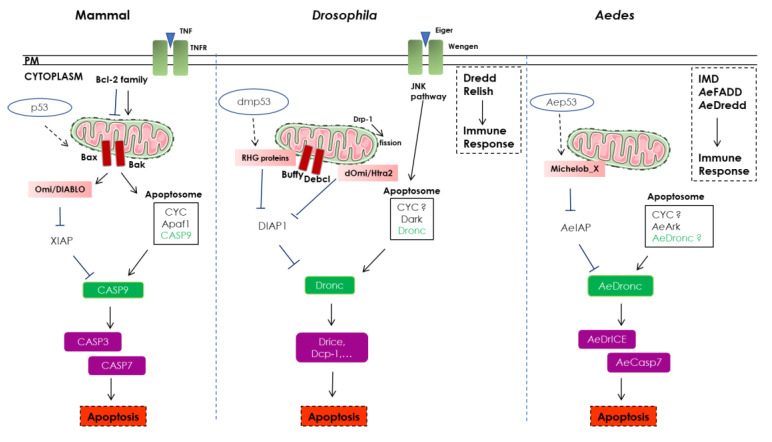

Recent knowledge concerning apoptosis in mosquitoes has been acquired through gene homology with *Drosophila melanogaster*. Apoptosis is under the control of initiator and effector caspases [162], expressed ubiquitously and synthesized as inactive procaspases. The Apaf-1-related killer (Ark) molecule [163] assembles itself into an apoptosome-like complex [164] to activate Dronc, but the role of CYC in the insect apoptosome is very controversial [165]. Two Bcl-2 orthologues have been identified in *Drosophila*: Buffy and Debcl [166], whose pro- or anti-apoptotic roles are not clear [166,167]. Finally, mitochondrial fission, through Drp1 activation, seems to be required for efficient cell death [168]. However, the pro-apoptotic activity of caspases is mainly regulated by members of insect IAP family [169,170]. IAP antagonists (dOmi/HtrA2 and RHG proteins in drosophila; Michelob_X in mosquito) are localized in the mitochondrial intermembrane space in living cells and released into the cytosol, but remain near the mitochondria, after an apoptotic stimulus [170,171], where they compete for caspase binding through their IAP-binding domain. Additionally, RHG proteins can induce DIAP1 ubiquitination and degradation [168,172]. Interestingly, RHG and Mx promoters present different response elements regulated by transcription factors, such as dmp53, activated by developmental or environmental signals, leading to cell death [173]. In insects, no clear distinction can be made between intrinsic or extrinsic apoptotic pathways. Nevertheless orthologues of TNF (Eiger) and TNFR (Wengen) have been described in *Drosophila* [174] and induce cell death through a JNK-mediated pathway, requiring apoptosome components [175]. Immune response against pathogens is triggered by the NF-κB (Relish) pathway induction through the Immune Deficiency IMD/dFADD/Dredd (CASP8) pathway [176] in *Drosophila* and IMD/AeFADD/AeDredd in *Aedes* [177].

Finally, autophagy-dependent cell death is finely controlled in insects, and its role in development has been largely studied in *Drosophila* [178,179]. To our knowledge, pyroptosis and necroptosis have not yet been described in insects.

Thus, caspase activity may be required for dissemination of SINV from the midgut to the secondary organs by participating in the remodeling of the basal lamina, as suggested in baculovirus-infected lepidopteran, where caspase and MMP activity is necessary to cross the midgut barrier [180]. However, cell death modulation in vitro, in mosquito cells, does not seem to alter alphaviral replication. In fact, recombinant SINVs, expressing Reaper (*Drosophila* IAP inhibitor) or Michelob_X (Mx, *Aedes* IAP inhibitor), induce apoptosis in infected *Aedes Albopictus* C636 cells, with no inhibitory effect on viral production in the initial phase of infection. Moreover, in these conditions, inhibition of caspase activity has no effect on viral replication neither [181]. However, recombinant SINV expressing Reaper induces cell death in vivo in *Aedes Aegypti*’s midgut, a delayed infection and propagation in the saliva [182]. More importantly, this last study also describes a rapid genetic selection of SINV variants in vivo against Reaper expression.

Hence, cell death, with tissue degradation features, seems important for alphavirus propagation in mosquito organism, with no clear effect on replication in vitro. However, as described above for mammal cells, RCD is also associated to efficient immune response against viruses and may be one of the key processes involved in mosquito resistance to virus.

How cell death is modulated in infected tolerant mosquito cells remains to be understood. Oxidative stress response may play an important role in the mosquito’s response. Indeed, CHIKV infection induces upregulation of antioxidant pathways in mosquito midgut, which delays cell death [183]. During arbovirus infection, oxidative stress is actually detected in both mammal [101,123,184] and insect cells. Oxidative stress is defined by loss of homeostasis between accumulation of ROS and production of antioxidant enzymes such as superoxide dismutase (SOD), catalase (CAT) or glutathione transferase and reductase [185]. After blood feeding, the midgut is in contact with sugar, iron, heme and other components of vertebrate blood. Mosquitoes have developed protective adaptation against the damage caused by heme and iron uptake. Indeed, the heme can induce lipid peroxidation, protein degradation, and ultimately cell death. Once in the epithelial cells, these components are detoxified, and a strong antioxidant and protective response is engaged [186]. Concomitantly, pathogens present in the blood could take advantage of this antioxidant response, blocking cell death, to infect and replicate into midgut epithelial cells.

The pro-survival pathway PI3k–Akt–mTOR may also be involved in insect tolerance. Indeed, in drosophila, activation of the PI3k–Akt–mTOR pathway is associated to an increase of SINV infection, potentially through apoptosis and autophagy inhibition and a more efficient cap-dependent translation of viral genome [187]. Finally, the role of autophagy seems to be limited in CHIKV infection of mosquito cells. Even infection induces autophagy in Aag2 cells, every pharmacological modulation of autophagy (inducer or blocker) leads to a replication increase in mosquito cells [188].

Hence, the sensitivity may depend on the better resistance of midgut cells to oxidative stress induced by viral infection, leading to a delayed cell death but the involvement of autophagy in these regulations remains to be understood.

**Figure 3:**
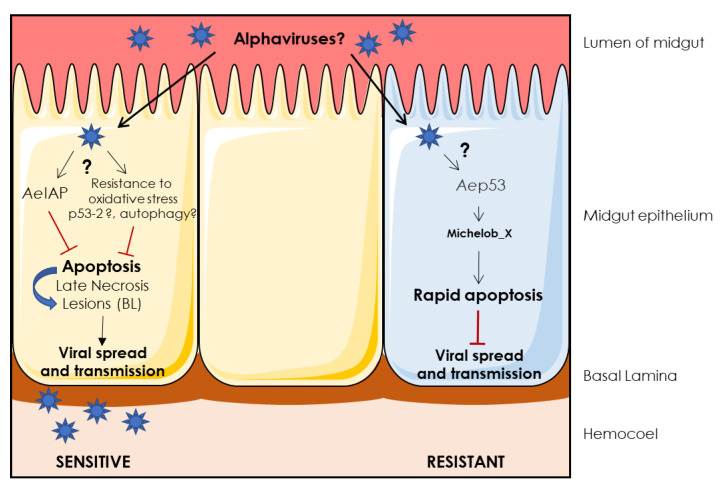
Possible interplay between cell death and alphavirus infection in *Aedes* mosquitoes: extrapolation from other arboviruses studies. BL: basal lamina, *Ae*IAP: *Aedes* inhibitor of apoptosis protein. See Box 1 for RCD pathway description in insect.

Few studies have been conducted to investigate the interplay between alphaviruses and their vector. However, recent findings in mosquito and drosophila underlie the role of p53 isoforms in cell response to oxidative stress and to DENV infection. The balance between a rapid apoptosis and a delayed, secondary necrosis may explain in part the differences between tolerant and resistant mosquitoes’ strains. For more details see [177,189,190,191,192,193,194,195,196] and Figure 3 for suggested mechanisms which may be involved in alphavirus infection in sensitive versus resistant mosquitoes, extrapolated from other arboviruses.

In addition to the virus’ ability to penetrate the cells of a particular mosquito (specific receptor, lipid membrane composition), the difference between resistant and tolerant strains may also lie in the rapidity of cell response to viral infection in the first targeted tissue, i.e., the mosquito gut. This response has to be apoptotic and not necrotic to ensure mosquito resistance. A p53/Mx- [189] and caspase- [177] dependent cell death has been linked to *Aedes Aegypti* resistance to DENV infection. Phagocytosis of dead cells and apoptotic bodies also seem to be important for a virus specific immune response, at least in drosophila [190].

Sensitive mosquitoes may tolerate viral infection through IAP-dependent apoptosis inhibition, as shown for the arbovirus bluetongue virus (BTV) [191,192]. Another tolerance mechanism to DENV seems to rely on resistance to oxidative stress through CAT protection in mosquito gut [193] or p53 isoforms regulation leading to cell survival in drosophila [194] and mosquito [195,196]. Delayed apoptosis may then lead to secondary necrosis, impairing a proper innate immune response, favoring basal lamina damage and viral spread [189].

## 7. Conclusions

The comparative overview of cell response to alphavirus infection, in mammals and mosquitoes, underlines the complexity of cell death regulation among different species facing the same pathogen. In both, host and vector, the first cells in contact with the virus will influence the progression of the infection, through immune response and cell death.

In mammals, alphavirus-induced apoptosis is also linked to pathogenesis in the organs secondarily infected. Some discrepancies between intrinsic and extrinsic apoptotic pathways in mammals could be explained by a secondary amplification loop through CASP6 activation or by a newly described CASP8 activation platform which directly links viral RNA sensors and apoptosis. However, the precise mechanisms need to be studied further, in other cell types and for different alphaviruses.

Experimentally interfering with apoptosis in vitro does not seem to influence the viral replication rate but can lead to persistence. It is possible that autophagy may be a way for alphaviruses to delay cell death, allowing replication over a longer period of time. Alphavirus non-structural proteins 2 and 3 appear to be mostly responsible for mammal cell death, which is not the case for mosquito cells from sensitive strains. Mammals are often considered to be an “accidental host” in arboviral infections, suggesting that nsp-induced cell death may be also “accidental” and in some way deleterious for alphaviruses. Some alphaviruses have indeed evolved to be restricted to mosquitos and do not rely on transmission to mammals any longer [197,198,199].

How tolerant mosquito cells survive to arbovirus infection remains unknown. In addition to a strong action of insect IAPs, a higher control of oxidative stress due to the fact of infection through p53 isoforms and autophagy may be an answer, but supplementary studies are needed for alphaviruses. Moreover, in the case of arthritogenic alphaviruses in mammals, clarification of the processes leading to chronic infection and a possible survival of infected cells is needed. The differences in cell types may explain these discrepancies and further studies would help to decipher how these cells overcome cell death.

Finally, even if it is generally admitted that alphavirus infection is silent in competent mosquitoes, a certain level of tissue destruction is observed and needed in gut epithelia and salivary glands to allow viral propagation and transmission. As alphaviruses are still poorly studied to understand the link between cell death and vector competence, we can only extrapolate from recent DENV studies. Indeed, in addition to immune response, the mosquito’s ability to rapidly eliminate infected epithelial cells through apoptosis, instead of a delayed cell death and secondary necrosis, may explain the difference between a resistant mosquito and a tolerant one. A better understanding and subsequent manipulation of vector tolerance could help to control arboviral propagation, as has recently been suggested [15,18].
